# Risk of Diabetes Mellitus and Its Association with Cardiorespiratory Endurance in Zenú Indigenous People in Momil, Córdoba, Colombia

**DOI:** 10.3390/ijerph23060811

**Published:** 2026-06-18

**Authors:** Leily Montoya-Alvarez, Edgar Rodriguez-Sepúlveda, Claudia Galeano-Páez, Osnamir Elias Bru-Cordero, Noelba Alcala-Tafur

**Affiliations:** 1 MOSABI Research Group, Master’s Programme in Epidemiology, University of Sinú, Montería 230001, Colombia; leilymontoya@unisinu.edu.co; 2 Master’s Programme in Epidemiology, University of Sinú, Montería 230001, Colombia; edgarfrodriguez@unisinu.edu.co; 3 Biomedical Research and Molecular Biology Group, University of Sinú, Montería 230001, Colombia; 4 Academic Affairs Office, National University of Colombia, La Paz Campus, Kilometre 9, Valledupar 2022010, Colombia; oebruc@unal.edu.co; 5 Physical Therapy Program, University of Sinú, Montería 230001, Colombia; noelbaalcala@unisinu.edu.co

**Keywords:** type 2 diabetes mellitus, risk factors, indigenous population, cardiorespiratory endurance

## Abstract

**Highlights:**

**Public health relevance—How does this work relate to a public health issue?**

- Type 2 diabetes mellitus represents a growing public health challenge worldwide and in Colombia, with a disproportionate impact on indigenous populations due to social inequities, limited access to preventive services, and a high prevalence of modifiable risk factors such as overweight, central adiposity, and physical inactivity.
- This study addresses a critical evidence gap by evaluating the risk of type 2 diabetes and its association with aerobic endurance in an underserved Zenú indigenous community, contributing population-level data relevant to chronic disease prevention in vulnerable groups.

**Public health significance—Why is this work of significance to public health?**

- The use of validated, low-cost, and non-invasive tools (FINDRISC and Ruffier test) demonstrates feasible strategies for early identification of metabolic risk in community settings, particularly in low-resource and culturally diverse populations.
- By characterizing diabetes risk profiles in an indigenous population undergoing epidemiological and nutritional transition, the study provides context-specific evidence to inform preventive health actions and community-based screening programs.

**Public health implications—What are the key implications or messages for practitioners, policy makers and/or researchers in public health?**

- Public health professionals and policymakers should prioritize culturally adapted prevention strategies focused on weight management, promotion of regular physical activity, and early risk screening among indigenous populations, particularly for individuals with a family history of diabetes and central obesity.
- For researchers, the findings highlight the need for longitudinal studies and the incorporation of more sensitive measures of cardiorespiratory fitness to better understand its role in metabolic risk and to strengthen evidence-based interventions in indigenous and other vulnerable populations.

**Abstract:**

Type 2 diabetes mellitus (T2DM) represents a growing public health concern in Indigenous populations, where early risk identification remains limited. This study aimed to analyze the association between estimated T2DM risk and cardiorespiratory endurance in a Zenú Indigenous community in northern Colombia. A cross-sectional study was conducted among adults aged 18–70 years belonging to the Momil Urban Minor Indigenous Cabildo (Córdoba, Colombia). T2DM risk was assessed using the Finnish Diabetes Risk Score (FINDRISC), and cardiorespiratory endurance was evaluated through the Ruffier index. Associations were examined using a multivariable ordinal logistic regression model, and discriminative performance was evaluated using receiver operating characteristic (ROC) curve analysis. A total of 95 participants were included, most of whom were classified as low risk according to FINDRISC, while cardiorespiratory endurance assessed by the Ruffier index was predominantly classified as moderate to good. Age was significantly associated with higher risk categories in the adjusted model, whereas cardiorespiratory endurance was not significantly associated with estimated T2DM risk. The model demonstrated excellent discriminative capacity (AUC = 0.912; 95% CI: 0.850–0.973). In this population, age and family history were key determinants of estimated T2DM risk. Findings regarding cardiorespiratory endurance should be interpreted cautiously given sample size limitations.

## 1. Introduction

Diabetes mellitus is a persistent metabolic disorder characterized by high concentrations of glucose in the bloodstream [1]. This condition is associated with the development of cardiovascular disease and additional health complications [2] and is one of the leading causes of death in people between the ages of 30 and 70 [3]. The American Diabetes Association (ADA) classifies diabetes into four types according to its etiology: T2DM, T1DM, gestational diabetes, and specific forms of diabetes derived from alternative factors [4].

Globally, T2DM is the most common type, accounting for 90–95% of all cases [5]. Its incidence has shown a sustained upward trend, with an age-adjusted rate of 618 cases per 100,000 inhabitants/year for 2021, representing an approximate increase of 51% compared to 1990 [6]. Furthermore, T2DM was responsible for 1.66 million deaths worldwide, with an age-adjusted mortality rate of 19.6 per 100,000 inhabitants, ranking as the eighth leading cause of death globally [7]. The increasing burden of T2DM is attributed to the interaction between genetic predisposition and environmental or behavioral factors, with variations observed across different demographic groups [8].

Among vulnerable populations, indigenous communities have been disproportionately affected by T2DM. According to the International Diabetes Federation (IDF) 2023 report, T2DM has become one of the most common health conditions impacting indigenous populations worldwide [9]. Between 2000 and 2018, studies conducted in Mexico and Guatemala documented a marked increase in diabetes incidence within indigenous communities, with higher prevalence rates compared to non-indigenous populations [10].

In Colombia, DM and cardiovascular diseases (CVD) represent major public health challenges, contributing significantly to morbidity and mortality among adults [11]. In 2022, 8703 deaths from T2DM were recorded, corresponding to a mortality rate of 16.5 per 100,000 population. Premature mortality (30–70 years) reached 13.3 deaths per 100,000 inhabitants, confirming T2DM as an important cause of preventable death in the productive-age population [12]. The reported prevalence varies according to region and access to medical care. Studies have reported a range between 1.84% and 11.2%. In rural areas, lower rates between 1.4% and 7.9% have been documented [13], while the prevalence in the general Colombian population is approximately 7.1% [14].

At the regional level, in the department of Córdoba, T2DM represents a growing public health concern influenced by social and territorial determinants [15]. Although Córdoba is not among the departments with the highest diabetes mortality rates in Colombia, the actual burden may be underestimated due to underreporting of underlying causes of death in rural areas and limited access to metabolic control services, particularly among vulnerable populations [16]. This situation reflects broader territorial inequalities, where peripheral departments experience higher premature mortality and reduced availability of specialized healthcare services compared to major urban centers [17]. The coexistence of overweight, sedentary lifestyles, and unfavorable socioeconomic conditions further increases the risk of adverse metabolic outcomes in the region [18].

Within this context, the Zenú indigenous population of the municipality of Momil (Córdoba) represents a community for which limited scientific evidence exists regarding T2DM risk and associated factors. Identifying risk factors—such as age, sex, obesity, hypertension, and genetic susceptibility—is essential, as these are associated with alterations in body mass index (BMI), waist circumference, and blood glucose levels [19]. Early identification of individuals at high risk is critical for preventing complications, particularly cardiovascular disease [20].

Cardiorespiratory endurance (CE) is a key component of physical fitness and a strong marker of cardiovascular health [21]. Higher CE levels are associated with a lower probability of developing CVD, hypertension, and T2DM [22]. Conversely, individuals with low CE have a 56% greater likelihood of developing CVD and a 70% higher risk of mortality [23].

Despite the documented burden of T2DM in indigenous populations, there is a lack of local studies analyzing the relationship between T2DM risk and components of physical fitness, such as CE, in the Zenú community of Momil, Colombia. This knowledge gap limits the development of culturally appropriate, evidence-based preventive strategies tailored to the sociocultural and environmental characteristics of this population. The Zenú community is an officially recognized indigenous group with its own governance structures and sociocultural practices, residing primarily in the municipality of Momil, Córdoba. These unique characteristics—including communal organization, traditional lifestyle practices, and geographic distribution—affect both access to health services and participation in research studies, highlighting the importance of investigating T2DM risk and cardiorespiratory endurance within this specific population.

Therefore, this study aims to analyze the association between the risk of T2DM and cardiorespiratory endurance in the Zenú indigenous population of the municipality of Momil, Córdoba, using a quantitative, analytical, cross-sectional design. The FINDRISC questionnaire was employed to estimate the 10-year risk of T2DM, and the Ruffier test was used to assess CE as an indicator of cardiovascular health.

## 2. Materials and Methods

### 2.1. Study Design

This is a quantitative, analytical, cross-sectional study with a non-experimental approach that seeks to establish the association between categorical variables, specifically between the potential risk of T2DM and CE levels in the Zenú indigenous population of the municipality of Momil, Córdoba, Colombia.

### 2.2. Study Area

This study was conducted in the municipality of Momil, located in the San Jorge subregion of the department of Córdoba, northern Colombia. The area forms part of the ancestral territory of the Zenú indigenous people, a community recognized for its socio-political organization, cultural identity, and close relationship with the river and wetland ecosystems that predominate in the region [10]. The municipality of Momil is characterized by an economy based on agriculture, livestock, crafts, and fishing, as well as a significant indigenous population distributed among legally constituted councils [13].

**Figure 1:**
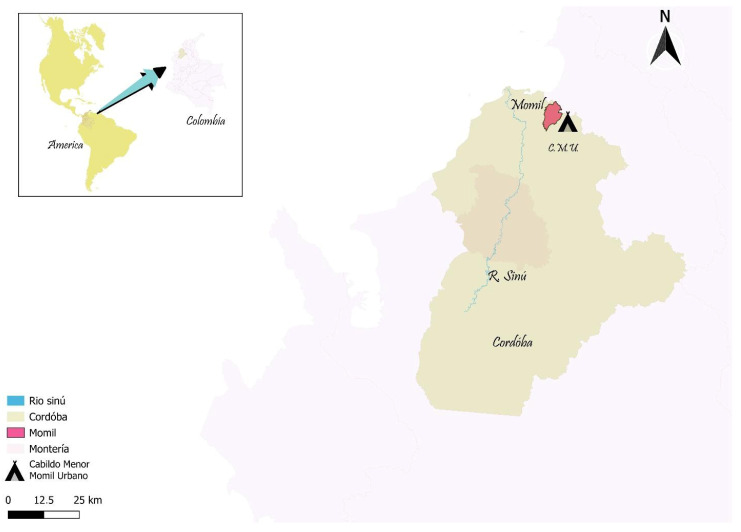
Geographic location of the municipality of Momil in the department of Córdoba, Colombia. The inset map indicates the location of the study area within the American continent (arrow). In the main map, Momil is highlighted in red within Córdoba, and the triangle indicates the location of the Momil Urban Minor Cabildo (C.M.U.), where the study was conducted.

The study area was limited to the Zenú de Momil indigenous reserve, specifically in communities that, although currently located in the urban center of the municipality, preserve their own cultural practices despite their urban location (Figure 1).

### 2.3. Ethical Aspects

The research project was approved by the Research Ethics Committee of the Faculty of Health at the University of Sinú–Elías Bechara Zainúm (Approval No. 001, Minutes of 4 March 2024). As this study followed a cross-sectional observational design, clinical trial registration was not required.

Likewise, written approval was obtained from the Board of Directors of the Indigenous Cabildo (a traditional Indigenous governing authority recognized under Colombian law). A formal document signed by the captain, as the highest authority, was provided as official authorization to use its headquarters as the site for data collection and assessment procedures. The research procedures and safeguards for participants were conducted in accordance with the ethical principles of respect, autonomy, justice, beneficence, and non-maleficence, and in compliance with the ethical guidelines established in Articles 5 and 6 of Resolution 8430 of 1993 issued by the Colombian Ministry of Health [24].

Individuals participated in the research on a voluntary basis, after signing an informed consent form setting out the purpose of the study, the measures to be taken, and the risks involved, ensuring that they understood everything contained in the document. Participants were also informed that they could withdraw from the study at any time during its course if they so wished.

### 2.4. Population and Sample

The study population consisted of adults aged 18 to 70 years belonging to the Momil Urban Minor Indigenous Cabildo, registered with the Ministry of the Interior, and residing in the municipality of Momil, Córdoba. Recruitment was conducted during different Cabildo meetings held at the main headquarters in Momil, which provided multiple opportunities for eligible individuals to participate. A non-probabilistic convenience sampling approach was used, selecting participants based on their accessibility and willingness to participate in the study. The following exclusion criteria were applied: Indigenous individuals with a prior medical diagnosis of type 2 diabetes mellitus (T2DM), those with diseases causing physical or functional limitations, and those who did not sign the informed consent form or who voluntarily declined participation.

To determine the appropriate sample size for this research, a total population of 150 individuals attending the fortnightly meetings held by the Cabildo was considered. Using a margin of error of 5% and a confidence level of 95%, the standard formula for calculating sample size in a finite population was applied, resulting in an estimated required sample of 108 participants. Ultimately, a total of 95 participants met the eligibility criteria and were included in the final analysis.

### 2.5. Data Collection

Before the instruments were applied, the purpose of the study was explained to each participant, and informed consent was obtained, guaranteeing the confidentiality of the information and voluntary participation. The interviews were conducted by the principal investigator with the support of trained assistants, following a standardized protocol to ensure uniformity in the application of the instruments. The first data collection instrument was a sociodemographic characterization questionnaire that included variables such as sex, age, socioeconomic status, educational level, area of residence, and type of affiliation to the EPS health system.

Subsequently, the FINDRISC questionnaire was applied, which has been previously validated by Soriguer et al. [25] and approved by the FID to detect the presence of risk of T2DM [26]. This instrument is widely used and validated in the Colombian and Latin American adult population, including regional adaptations (LA-FINDRISC) that adjust anthropometric cut-off points for Hispanic and mestizo contexts [27]. However, it is important to note that, although FINDRISC has performed well in identifying dysglycemia and high risk of T2DM in Colombian adults, there are no formal reports of specific validation in Zenú indigenous communities or other indigenous communities in the Colombian Caribbean. Therefore, in this study, FINDRISC was used as a population screening tool, recognizing that risk cut-off points may require adjustments in the future to adequately capture the morphological, nutritional, and sociocultural particularities of this community [28]. In addition, the survey demonstrates a sensitivity rate of 81% and a specificity rate of 76% in risk prediction using eight non-invasive clinical parameters [29], including age, BMI, abdominal circumference, physical activity, consumption of fruit and vegetables, history of high blood glucose levels, use of medication for hypertension, and family history of diabetes (Table 1).

No additional sociocultural variables beyond those included in the FINDRISC questionnaire were independently assessed. However, for the items related to physical activity and consumption of fruits and vegetables, responses were interpreted considering the participants’ traditional lifestyle practices and customary dietary patterns within the community context.

**Figure 2:**
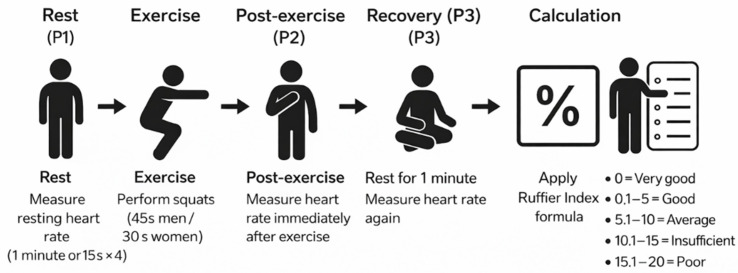
Infographic of the procedure and classification of the Ruffier test.

Next, CE was measured using the Ruffier test, which aims to measure cardiorespiratory fitness response to short-duration exertion and cardiac recovery capacity, and therefore the level of physical fitness of the heart [30], by performing the following steps:

The procedure consists of six stages (Figure 2). First, the resting heart rate (P1) is recorded for one minute (or for 15 s multiplied by four). Next, leg flexion-extension exercises are performed with the trunk straight. At the end of the exercise, the post-exercise heart rate (P2) is measured immediately. After one minute of recovery at rest, the heart rate is recorded again (P3). These three values are used to calculate the Ruffier index, which classifies physical performance into five levels: very good (0), good (0.1–5), average (5.1–10), insufficient (10.1–15), and poor (15.1–20; requires medical evaluation).

### 2.6. Data Analysis

Data were cleaned, checked for consistency, and examined for outliers. A descriptive analysis of missing data was conducted to assess the completeness of the dataset and determine the proportion of incomplete observations for each variable. Participants who did not meet the inclusion criteria or had incomplete critical information were excluded from the final dataset. Subsequently, the data were organized and tabulated in Microsoft Excel and analyzed using R software [31]. The analysis began with a descriptive assessment of sociodemographic variables, FINDRISC components, and aerobic capacity (Ruffier index), applying measures of central tendency, dispersion, and proportions according to the nature of the variables. Normality was assessed using the Shapiro–Wilk test, which indicated a non-normal distribution; therefore, the Kruskal–Wallis test was applied to compare FINDRISC risk categories. A multivariable ordinal logistic regression model (proportional odds model) was fitted to identify factors associated with FINDRISC risk categories (low, moderate, high). Candidate variables included age, sex, anthropometric indicators, aerobic capacity (Ruffier index), and family history of diabetes. Model selection was guided by the Akaike Information Criterion (AIC), and age and family history of diabetes were retained in the final adjusted model based on parsimony and model fit. Family history was coded dichotomously (yes/no). The proportional odds assumption was formally evaluated and verified. A 95% confidence level and a statistical significance threshold of *p* < 0.05 were established.

## 3. Results

### 3.1. Sociodemographic Characteristics

The voluntary participation of 95 people who met the established inclusion criteria was achieved. Of these, 76.8% were women and 23.2% were men. The average age was 46.5 ± 14.1 years, with the highest concentration in the under-45 (38.9%) and 45–54 (28.4%) age groups. In terms of educational level, almost half had completed secondary school (47.4%), followed by higher education (30.5%) and basic education (22.1%). Most participants (93.7%) resided in urban areas.

The mean BMI of the population was 27.2 ± 4.0 kg/m^2^ (Table 2). According to BMI category classification, 51.6% of the participants were overweight, 20.0% had grade I obesity, and 3.2% had grade II obesity. Only 24.2% were within the normal weight range, and 1.1% were underweight.

When analyzing the results by sex, overweight predominated in women (52.1%), followed by grade I obesity (26.0%). In men, the most frequent category was also overweight (50.0%), followed by normal weight (45.5%). No cases of grade I obesity were recorded in the male group, and grade II obesity was not observed in either sex.

Overall, 74.8% of the sample presented excess weight (overweight or obesity).

### 3.2. Diabetes Risk Levels vs. Characteristics Measured by FINDRISC

Table 3 presents the results of the risk level for developing T2DM, classified according to the dimensions assessed by the questionnaire, such as sociodemographic and anthropometric characteristics and lifestyle habits of the study population.

Age showed an increasing trend across risk categories, although no statistically significant association was observed (*p* = 0.38). BMI was significantly associated with FINDRISC risk level (*p* = 0.03), with a lower proportion of low-risk individuals among those with obesity (BMI > 30 kg/m^2^). Waist circumference also showed a statistically significant association (*p* = 0.003).

Personal history of hyperglycemia (*p* < 0.001) and family history of diabetes (*p* < 0.001) were significantly associated with higher FINDRISC risk levels.

### 3.3. Aerobic Endurance (Ruffier Test)

Among the 95 participants assessed using the Ruffier index, aerobic capacity was distributed as follows: good (56.8%), average (37.9%), very good (4.2%), and insufficient (1.1%) (Table 4), with 98.9% performing at least at an average level or above.

Table 5 shows that participants with moderate risk had higher resting and post-exercise heart rates, accompanied by a lower percentage recovery, which could reflect lower cardiorespiratory efficiency. In contrast, the low-risk group showed a more pronounced physiological increase during exercise and more efficient recovery, consistent with better CE.

### 3.4. Comparison of Clinical Parameters (Weight and BMI) According to FINDRISC Risk Level

**Figure 3:**
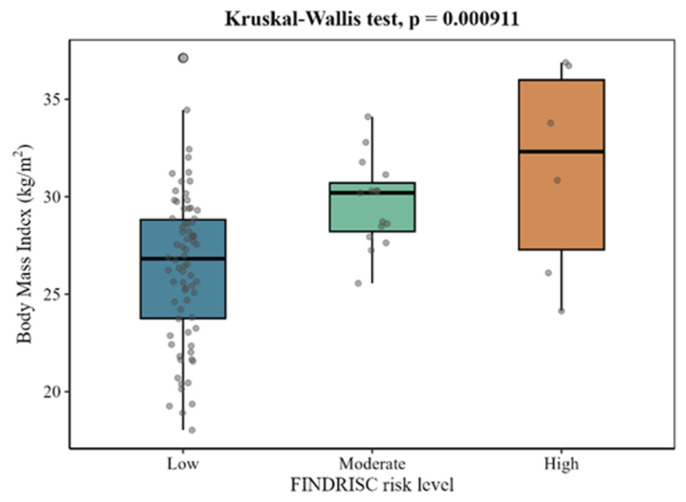
Kruskal–Wallis test of BMI vs. FINDRISC.

Figure 3 illustrates the comparison of the BMI variable among the different T2DM risk levels established by the FINDRISC test (low, moderate, and high), showing the anthropometric variations associated with increased risk.

Statistically significant differences in BMI were observed according to risk level (Kruskal–Wallis test, *p* = 0.00091), showing an increasing trend in BMI as the estimated risk of T2DM increased.

### 3.5. Age, Family History, and FINDRISC Risk

A multivariable proportional odds ordinal logistic regression model was fitted to estimate the association of age and family history of diabetes with FINDRISC risk categories (low, moderate, high). The detailed regression results are presented in Table 6.

Age was independently associated with higher FINDRISC risk categories, indicating that increasing age was related to greater odds of belonging to a higher risk level. Although family history of diabetes showed a positive association with increased risk, this relationship did not reach statistical significance in the adjusted model.

**Figure 4:**
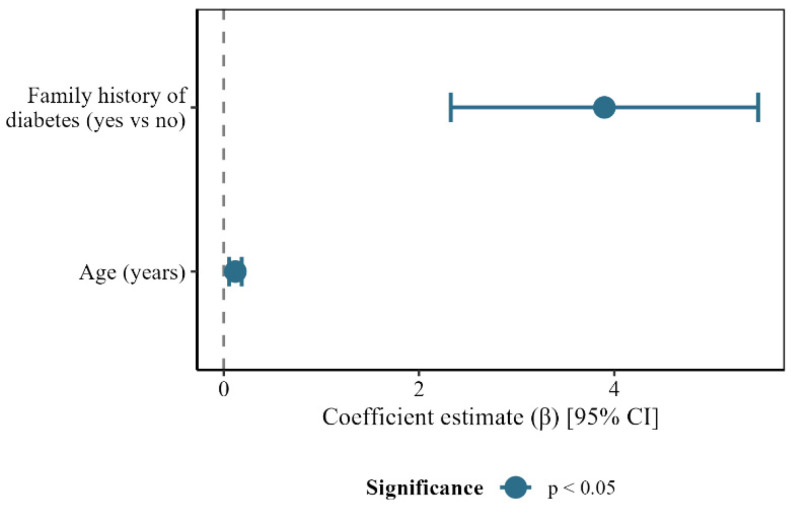
Effect of age + family history of diabetes on FINDRISC.

Pairwise comparisons between outcome thresholds indicated a significant distinction between the low- and moderate-risk categories, whereas no statistically significant difference was observed between the low- and high-risk categories (Figure 4).

Figure 4 illustrates the estimated effects of age and family history of diabetes on the probability of transitioning across FINDRISC risk categories under the proportional odds assumption.

**Figure 5:**
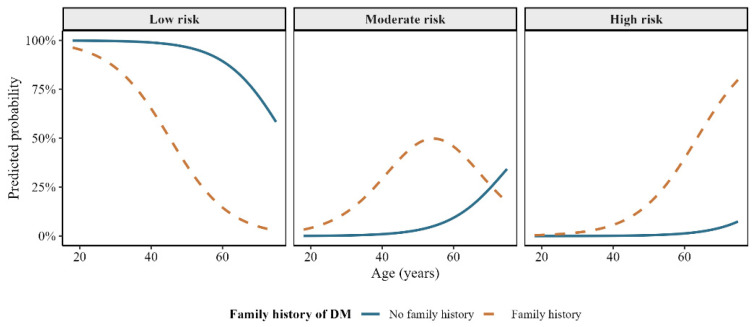
Adjusted FINDRISC model according to age and family history of diabetes.

Figure 5 presents predicted probabilities derived from the adjusted ordinal logistic regression model. Among individuals without a family history of diabetes, the probability of remaining in the low-risk category remained high (>0.90) across the observed age range. In contrast, among those with a family history, the probability of low risk progressively decreased with increasing age.

The probability of moderate risk increased with age in both groups, with a steeper gradient among individuals with a family history of diabetes, demonstrating an earlier shift toward the moderate-risk category beginning in the third decade of life.

**Figure 6:**
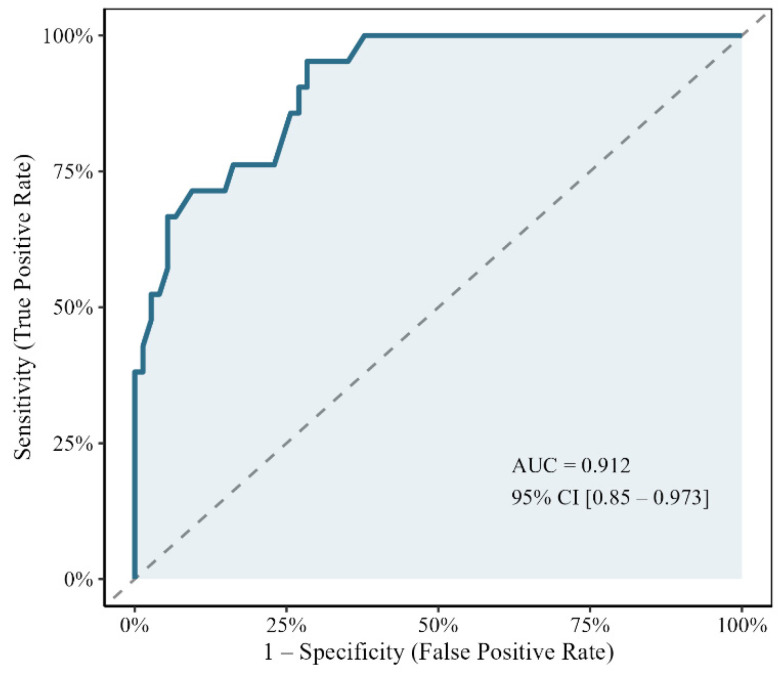
ROC curve—Ordinal logistic regression models (Low risk vs. Moderate/High risk; FINDRISK).

In Figure 6, the discriminative performance of the ordinal logistic regression model was evaluated through receiver operating characteristic (ROC) curve analysis, contrasting participants classified as low risk against those at moderate or high risk of developing T2DM according to the FINDRISC score. The model yielded an area under the curve (AUC) of 0.912 (95% CI: 0.850–0.973), indicating excellent discriminative capacity. This value suggests that the combination of age and family history of diabetes correctly distinguishes between risk categories in more than 91% of cases, substantially exceeding the threshold of 0.80 conventionally regarded as indicative of strong predictive performance. The model demonstrated excellent discriminative performance, with an area under the ROC curve (AUC) of 0.912 (95% CI: 0.850–0.973). The width of the confidence interval, while reflecting the constraints of a modest sample size, remains entirely above 0.80, reinforcing the robustness of the model’s classification ability. These findings position age and family history as clinically meaningful predictors of elevated T2DM risk within this Indigenous community and support the potential utility of this parsimonious model as a screening tool in resource-limited settings where more complex diagnostic approaches are not routinely available.

### 3.6. Association Between CE (Ruffier) and the Risk of Developing T2DM (FINDRISC)

**Figure 7:**
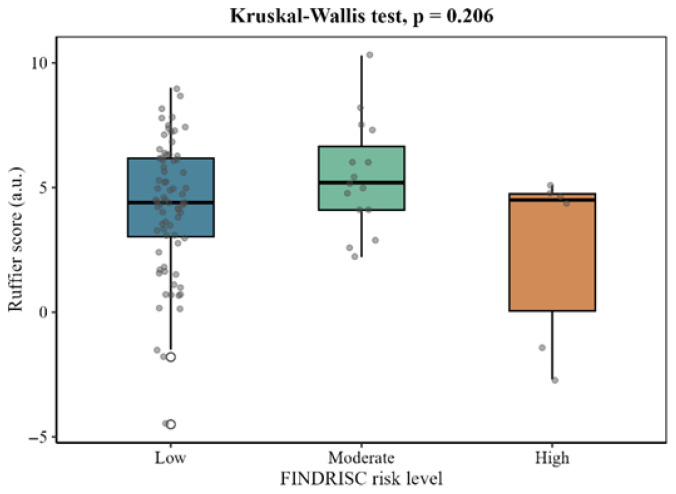
Kruskal–Wallis test between aerobic endurance and FINDRISC risk levels.

Figure 7 presents a comparison of the Ruffier test scores between the different levels of T2DM risk estimated by the FINDRISC test. No statistically significant differences in Ruffier scores were observed across FINDRISC risk levels. A slight tendency toward higher scores (indicative of lower CE) was observed in the moderate-risk group.

## 4. Discussion

The present study explored the association between estimated T2DM risk and CE in a Zenú indigenous community. Although most participants were classified as low risk according to FINDRISC, anthropometric indicators and familial history emerged as key determinants of risk stratification, while cardiorespiratory endurance assessed through the Ruffier index was not significantly associated with estimated T2DM risk.

The predominance of adult women with medium educational level reflects the demographic structure of the assessed community; however, given the cross-sectional design, these characteristics should be interpreted as contextual rather than causal. Previous evidence in Latin American indigenous populations has shown that older age, female sex, and lower educational attainment are associated with increased T2DM risk, likely mediated by lifestyle factors, adiposity patterns, and reduced access to preventive care [7].

Although educational level and residence were not the primary focus of this study, these contextual factors may still play an important role in shaping diabetes risk through broader social determinants of health, influencing prevention, management, and the risk of T2DM through differences in health literacy, access to healthcare services, and other social determinants of health. Previous research has shown that socioeconomic disadvantage, including lower educational attainment, is associated with higher diabetes prevalence and poorer quality of care in deprived areas, likely due to reduced access to information, healthy foods, and preventive services [32]. Furthermore, disparities between rural and urban areas have been reported in diabetes prevalence, with differences partly explained by sociodemographic characteristics and access to health resources [33]. In rural populations, limited awareness and lower education levels have been associated with lower diabetes knowledge and reduced utilization of preventive care [34]. These findings suggest that, even though educational level and residence area did not retain statistical significance in our adjusted model, they remain relevant social determinants that should be explored in future research with larger samples or alternative study designs.

Structural barriers—including geographic distance to health services, limited culturally adapted programs, and insufficient continuity of chronic disease management—may contribute to delayed diagnosis and suboptimal metabolic control in indigenous populations [23]. These contextual determinants are relevant when interpreting risk distribution patterns.

In the San Jorge subregion, where the Zenú community of Momil is located, previous evidence has documented structural inequalities affecting access to health insurance and specialized care among Indigenous populations [35]. Limited coverage of chronic disease prevention programs, cultural patterns influencing dietary habits and physical activity, and geographic and administrative barriers to healthcare access may contribute to delayed diagnosis and poorer metabolic control. Although the Momil Urban Cabildo is geographically situated within the municipal urban center, these communities experience structural vulnerability comparable to rural settings, particularly in relation to social determinants of health and ongoing epidemiological transition [36]. These contextual conditions are relevant when interpreting the distribution of metabolic risk observed in the present study.

Consistent with these contextual determinants, the high prevalence of excess weight underscores the central role of adiposity in metabolic risk. Both BMI and waist circumference showed significant associations with FINDRISC levels, reinforcing the importance of central adiposity in insulin resistance and metabolic dysfunction [37]. Similar associations have been documented in Australian and Ecuadorian indigenous populations [38,39] and are supported by evidence linking visceral adipose tissue to chronic low-grade inflammation and impaired insulin signaling [36].

It is important to consider that anthropometric indicators may not have identical metabolic implications across ethnic groups. Differences in body composition, fat distribution, and lifestyle patterns may influence the relationship between BMI, waist circumference, and cardiometabolic risk. Therefore, the application of standard international cut-off points may not fully capture risk profiles in specific indigenous populations. Future research should explore whether population-specific calibration or adapted anthropometric thresholds could improve risk classification accuracy in Zenú and other indigenous communities undergoing nutritional transition.

Although age did not reach statistical significance in the adjusted model, a progressive increase in risk across age groups was observed. This differs partially from multicenter Latin American analyses, where risk increases significantly with age [9]. Additionally, earlier onset of T2DM has been documented in Canadian indigenous communities [40], suggesting that nutritional transition and early metabolic exposure may modify conventional epidemiological patterns [38,41].

Personal history of hyperglycemia and family history of diabetes were strongly associated with higher risk. Previous hyperglycemia represents a robust predictor of future T2DM [42], while familial aggregation increases risk two- to four-fold when first-degree relatives are affected [43], reflecting combined genetic and environmental influences.

With respect to physical fitness, no statistically significant association was detected between cardiorespiratory endurance and estimated T2DM risk in this sample. However, this finding should be interpreted as lack of evidence of association rather than evidence of absence. The cross-sectional design precludes temporal inference, and the limited number of participants in the high-risk category may have reduced statistical power to detect subtle relationships. In addition, the Ruffier test, while practical for field assessment, may have limited sensitivity to capture nuanced differences in cardiorespiratory fitness compared to laboratory-based measurements.

Furthermore, the relatively homogeneous aerobic performance observed in the sample may have restricted variability, thereby limiting the capacity to identify discriminative gradients across risk categories. Therefore, the null findings should be interpreted cautiously, particularly considering the consistent association between reduced cardiorespiratory fitness and increased cardiometabolic risk reported in previous studies [44,45]. Future research using longitudinal designs and more precise physiological measurements is warranted.

When interpreting the discriminative performance of the adjusted ordinal logistic regression model, it is important to note that validation studies of FINDRISC and its modified versions in Latin American populations have generally reported more moderate AUC values. For example, large-scale applications of FINDRISC in Latin American and Caribbean populations have shown heterogeneous but typically moderate discrimination levels [37]. Similarly, external validation studies of the FINDRISC in Venezuela (EVESCAM study) reported acceptable but lower discriminative performance compared to the original Finnish cohort [46]. More recent evaluations in Peruvian healthcare workers and other regional samples have reported AUC values ranging approximately between 0.68 and 0.75 for detecting undiagnosed dysglycemia or T2DM [28,47]. In this context, the AUC of 0.912 observed in the present study suggests particularly strong discriminative capacity within this specific indigenous community. Nevertheless, this performance should be interpreted cautiously given the modest sample size and the limited number of participants in the high-risk category, and external validation in comparable indigenous populations would be necessary before considering broader implementation as a screening strategy.

During data collection, recruitment was restricted to individuals attending Cabildo meetings, as community regulations and ethical safeguards did not permit direct access to members’ residential or personal information, nor home visits without prior consent. Consequently, individuals residing outside the urban center or those not regularly attending meetings may not have been represented in the sample. These access constraints may have contributed to selection bias, and the included participants may not fully represent the broader Zenú Indigenous population in the municipality of Momil, thereby partially limiting the external validity and generalizability of the findings.

Although an initial sample size was calculated, the final number of participants analyzed was smaller than estimated, and the high-risk FINDRISC category included a limited number of individuals. This reduced sample size may have affected statistical power, particularly for detecting associations between cardiorespiratory endurance and estimated T2DM risk; therefore, non-significant findings should be interpreted with caution.

Risk estimation was conducted exclusively using the FINDRISC scale. Although a high discriminative performance of the adjusted model was observed in the present study, the FINDRISC instrument has not been formally validated in Zenú or Caribbean Indigenous populations. Therefore, external validation and formal assessment of calibration would be necessary before considering broader implementation in similar contexts. Additionally, the instrument does not incorporate other variables described in the literature as relevant predictors of diabetes development, which may have influenced risk estimation in this population.

Finally, some variables were based on self-reported information, which may have introduced information bias. Nevertheless, the findings provide relevant preliminary evidence in a little-studied Indigenous population and may serve as a foundation for future research using probabilistic sampling designs, external validation of risk models, and longitudinal follow-up incorporating objective metabolic measurements.

## 5. Conclusions

This study evaluated the association between estimated type 2 diabetes mellitus (T2DM) risk and cardiorespiratory endurance in a Zenú Indigenous community in Momil, Córdoba, Colombia. Most participants were classified as having low estimated risk according to the FINDRISC questionnaire, while higher risk scores were mainly associated with increasing age, family history of diabetes, and higher BMI. No significant association was identified between cardiorespiratory endurance and estimated T2DM risk. These findings highlight the relevance of established metabolic risk factors and provide baseline evidence to inform future research and culturally appropriate diabetes prevention strategies in Indigenous populations.

## Figures and Tables

**Table 1:** Variables evaluated and scoring system for the diabetes risk scale.

Variable	Categories	Score
Age (years)	<45	0
	45–54	2
	55–64	3
	>64	4
Body Mass Index (BMI)–(kg/m^2^)	<25	0
	25–30	1
	>30	3
Abdominal circumference (cm)	Men < 94 Women < 80	0
	Men 94–102 Women 80–88	3
	Men > 102 Women > 88	4
Physical activity (≥30 min/day)	Yes	0
	No	2
Consumption of fruit and vegetables	Daily	0
	Not daily	2
Antihypertensive medication	Yes	2
	No	0
History of high blood glucose	Yes	5
	No	0
Family history of diabetes	None	0
	Grandparents, uncles, cousins, or siblings	3
	Parents, siblings, or children	5
Classification according to final score	low risk level (1% to 4% risk)	7–11
	Moderate risk level (17% risk)	12–14
	High risk level (33% to 50% risk)	>15

**Table 2:** Distribution of sociodemographic and contextual variables of the Zenú indigenous population by gender.

Variable	Category	Female n (%)	Male n (%)	Total n (%)
Age (years)	Mean ± SD	46.5 ± 14.1		
	<45	29 (39.7)	8 (36.4)	37 (38.9)
	45–55	24 (32.9)	8 (36.4)	31 (32.6)
	56–64	17 (23.3)	5 (22.7)	22 (23.2)
	>64	3 (4.1)	1 (4.5)	4 (4.2)
Educational level	Basic (no schooling, preschool, or primary school)	17 (23.3)	4 (18.2)	21 (22.1)
	Secondary (secondary school)	34 (46.6)	11 (50.0)	45 (47.4)
	Higher (technical, technological, university, or postgraduate)	22 (30.1)	7 (31.8)	29 (30.5)
Residence	Urban	69 (94.5)	20 (90.9)	89 (93.7)
	Rural	4 (5.5)	2 (9.1)	6 (6.3)
BMI categories (kg/m^2^)	Mean ± SD	27.2 ± 4.0		
	Underweight (<18.5 kg/m^2^)	1 (1.4)	0 (0.0)	1 (1.1)
	Normal weight (18.5–24.9 kg/m^2^)	13 (17.8)	10 (45.5)	23 (24.2)
	Overweight (25.0–29.9 kg/m^2^)	38 (52.1)	11 (50.0)	49 (51.6)
	Obesity class I (30.0–34.9 kg/m^2^)	19 (26.0)	0 (0.0)	19 (20.0)
	Obesity class II (35.0–39.9 kg/m^2^)	2 (2.7)	1 (4.5)	3 (3.2)
	Obesity class III (≥40.0 kg/m^2^)	0 (0.0)	0 (0.0)	0 (0.0)
Total		73 (76.8)	22 (23.2)	95 (100)

Note: Results are presented as absolute frequencies and percentages. Age and body mass index (BMI) are expressed as mean ± standard deviation. Percentages were calculated by column. Abbreviations: SD, standard deviation; BMI, body mass index; kg/m^2^, kilograms per square meter.

**Table 3:** Distribution of sociodemographic and lifestyle variables by level of risk for type 2 diabetes mellitus (FINDRISC).

Variables	Total (n)	Diabetes Risk Levels According to FINDRISC Scores			
		Low Risk (%)	Moderate Risk (%)	High Risk (%)	*p*-Value *
Age (years)	46.5 ± 14.0				0.38
<45	37	91.8	5.41	2.7	
45–54	32	75	15.63	9.37	
55–64	22	59.09	31.81	9.1	
>64	4	25	25	0	
Gender					
Female	73	75.34	17.81	6.85	0.54
Male	22	86.36	9.09	4.55	
BMI (kg/m^2^)	27.2 ± 4.03				0.03 *
<25	24	95.83	0.0	4.17	
25–30	49	83.67	14.29	2.04	
>30	22	45.45	36.36	18.18	
Waist circumference (cm)	89.0 ± 9.88				0.003 *
<94 (M), <80 (F)	27	100	0	0	
94–102 (M), 80–88 (F)	29	86.21	10.34	3.45	
>102 (M), >88 (F)	39	56.41	30.77	12.82	
Physical activity 30 min per day					0.95
Yes	37	78.38	16.22	5.41	
No	59	77.59	15.52	6.90	
Daily consumption of vegetables and fruit					0.52
Every day	52	78.85	17.31	3.85	
Not every day	43	76.74	13.95	9.30	
Regular use of antihypertensive medication					0.13
No	87	80.46	13.79	5.75	
Yes	8	50.0	37.50	12.5	
History of high blood glucose levels					<0.001 *
No	89	80.9	16.85	2.25	
Yes	6	33.33	0	66.67	
Family history of diabetes					<0.001 *
No	59	93.22	6.78	0	
Yes, second-degree relative	19	68.42	26.32	5.41	
Yes, first-degree relative	17	35.29	35.29	29.41	

Note: Results are presented as absolute frequencies and percentages. The *p*-values correspond to group comparisons performed using the chi-square test to assess the statistical association between each variable and the total FINDRISC risk score. Statistical significance was set at *p* < 0.05 (* indicates statistical significance).

**Table 4:** Cardiorespiratory endurance (Ruffier) by age and sex.

Ruffier Performance	Age (Years)												Total
	<45			45–54			55–64			≥65			
	F	M	Total	F	M	Total	F	M	Total	F	M	Total	
Very good	0	0	0	1	2	3	1	0	1	0	0	0	4
Good	18	6	24	12	1	13	11	4	15	1	1	2	54
Average	9	2	11	11	6	17	6	0	6	2	0	2	36
Insufficient	1	0	1	0	0	0	0	0	0	0	0	0	1
Total	28	8	36	24	9	33	18	4	22	3	1	4	95

Note: Results are presented as absolute frequencies. F, female; M, male. Age groups are expressed in years. Ruffier performance categories were classified as very good, good, average, and insufficient according to the Ruffier index criteria.

**Table 5:** Physiological parameters of heart rate in the phases of the Ruffier test according to FINDRISC risk level.

FINDRISC Risk Level	n	(HR_1_) Mean ± SD (lpm)	(HR_2_) Mean ± SD (lpm)	(HR_3_) Mean ± SD (lpm)	Increase (%)	Recovery (%)
Low risk	74	71.97 ± 9.26	93.01 ± 10.54	77.35 ± 10.97	+29.2%	−16.8%
Moderate risk	15	77.07 ± 6.92	95.07 ± 10.28	82.27 ± 10.62	+23.3%	−13.5%
High risk	6	69.67 ± 10.42	84.17 ± 12.70	70.83 ± 12.46	+20.8%	−15.7%

Note: HR_1_: resting heart rate (pre-exercise); HR_2_: heart rate immediately after exercise; HR_3_: heart rate after one minute of recovery. Increase (%) = ((HR_2_ − HR_1_)/HR_1_) × 100 and recovery (%) = ((HR_2_ − HR_3_)/HR_2_) × 100. Values are expressed in beats per minute (bpm).

**Table 6:** Multivariable proportional odds ordinal logistic regression model for FINDRISC risk categories.

Variable	β	Adjusted OR	95% CI	*p*-Value
Age (years)	0.039	1.04	1.00–1.08	0.041
Family history of diabetes (yes vs. no)	0.82	2.27	0.78–6.60	0.133

Note: β = regression coefficient; OR = odds ratio; CI = confidence interval. Adjusted ORs were obtained from a multivariable proportional odds ordinal logistic regression model including age and family history of diabetes. Model selection was guided by the Akaike Information Criterion (AIC). The proportional odds assumption was tested and verified. A 95% confidence level and a statistical significance threshold of *p* < 0.05 were applied.

## Data Availability

The data presented in this study are available from the corresponding author upon reasonable request. The datasets are not publicly available due to ethical and privacy restrictions, as they contain sensitive personal information from members of the Zenú indigenous cabildo who participated in the study.

## Abbreviations

The following abbreviations are used in this manuscript:

DM	Diabetes Mellitus
T1DM	Type 1 diabetes mellitus
T2DM	Type 2 diabetes mellitus
ADA	American Diabetes Association
CVD	Cardiovascular Diseases
IFD	International Federation of Diabetes
CE	Cardiorespiratory endurance
FINDRISC	Finnish diabetes risk score
BMI	Body mass index

## References

[1] Aleidi S.M., Al Fahmawi H., Masoud A., Rahman A.A. (2023). Metabolomics in Diabetes Mellitus: Clinical Insight. Expert Rev. Proteomics.

[2] OECD (2023). Health at a Glance: Latin America and the Caribbean. https://www.oecd.org/en/publications/health-at-a-glance-latin-america-and-the-caribbean-2023_532b0e2d-en.html.

[3] Minsalud En El Día Mundial de La Diabetes: MinSalud Promueve Prácticas de Vida Saludable. https://www.minsalud.gov.co/Paginas/En-el-Dia-Mundial-de-la-Diabetes-MinSalud-promueve-praticas-de-vida-saludable.aspx.

[4] American Diabetes Association (2021). 2. Classification and Diagnosis of Diabetes: Standards of Medical Care in Diabetes-2021. Diabetes Care.

[5] Goyal R., Singhal M., Jialal I. (2023). Type 2 Diabetes. StatPearls [Internet].

[6] Yu X., Kan C., Zhang K., Zhang X., Ren J., Chen J., Wang Y., Zhang Y., Zhang G., Sun X. (2025). Global Epidemiology and Burden of Type 2 Diabetes in Adults Aged 55 and Older: Insights from 1990 to 2021. Ther. Adv. Endocrinol. Metab..

[7] World Health Organization Estimaciones de Mortalidad y Salud Mundial. https://www.who.int/data/gho/data/themes/mortality-and-global-health-estimates?utm_source.

[8] Buichia-Sombra F.G., Dórame-López N.A., Miranda-Félix P.E., Castro-Juarez A.A., Esparza-Romero J. (2020). Prevalence and factors associated with type 2 diabetes mellitus in the indigenous population of Mexico: Systematic review. Rev. Medica Inst. Mex. Seguro Soc..

[9] International Diabetes Federation (2022). Diabetes Atlas Report on Diabetes Among Indigenous Peoples.

[10] Castro-Porras L.V., Rojas-Martínez R., Romero-Martínez M., Aguilar-Salinas C.A., Escamilla-Nuñez C. (2023). The Trend in the Prevalence of Diabetes Mellitus in the Mexican Indigenous Population from 2000 to 2018. AJPM Focus.

[11] Clavijo C., Medina M.T., Cortés D., Varela D., Bedoya L., Velásquez V.L., Maturana D., Hernández E., Polanco J.P., Revelo R.R. (2025). Análisis de la asociación entre el riesgo de diabetes y el riesgo cardiovascular en una población colombiana: Resultados basados en las escalas de la Findrisk y la OPS. Rev. Colomb. Endocrinol. Diabetes Metab..

[12] DANE (2023). Estadísticas Vitales Cifras de Defunciones.

[13] Bauer H., Concha Mendoza G.A., Kreienbrock L., Hartmann M., Frickmann H., Kann S. (2022). Prevalence of Common Diseases in Indigenous People in Colombia. Trop. Med. Infect. Dis..

[14] Acosta Ruiz L.X., Merchán M.A., Orjuela Vargas L., Acosta Ruiz L.X., Merchán M.A., Orjuela Vargas L. (2023). Diabetes mellitus tipo 2: Latinoamérica y Colombia, análisis del último quinquenio. Rev. Med..

[15] Ministerio de Salud y Protección Social (2024). Análisis de Situación de Salud Colombia 2023.

[16] Ministerio de Salud y Protección Social (2024). Indicadores Basicos en Salud.

[17] Organización Panamericana de la Salud Indicadores Básicos. https://www.paho.org/es/evidencia-e-inteligencia-para-accion-salud/indicadores-basicos-2023.

[18] Cuenta de Alto (2024). Costo Día Mundial de la Diabetes 2024.

[19] Brzozowska M.M., Havula E., Allen R.B., Cox M.P. (2019). Genetics, Adaptation to Environmental Changes and Archaic Admixture in the Pathogenesis of Diabetes Mellitus in Indigenous Australians. Rev. Endocr. Metab. Disord..

[20] Granda E.Á., Peláez D.M.A., Bravo A.S.A., Pitisaca E.E.E. (2025). Factores de riesgo asociados a la presencia de diabetes mellitus II en la población Shuar del cantón Macas. Cienc. Lat. Rev. Científica Multidiscip..

[21] Schroeder E.C., Franke W.D., Sharp R.L., Lee D. (2019). Comparative effectiveness of aerobic, resistance, and combined training on cardiovascular disease risk factors: A randomized controlled trial. PLoS ONE.

[22] Parra L.G.R., Jara J.L.G., Cando L.E.C., Tapia C.B.M. (2024). Impacto del entrenamiento deportivo en la salud cardiovascular. Tesla Rev. Científica.

[23] Ehsan F., Asim M. (2023). Assessment of Cardiorespiratory Fitness by the Ruffier Dickson Test and Its Correlation with Lifestyle Related Factors: A Cross Sectional Study among Pakistani Youth. J. Pak. Med. Assoc..

[24] Ministerio de Salud (1993). Resolución Número 8430 DE 1993.

[25] Soriguer F., Valdés S., Tapia M.J., Esteva I., Ruiz de Adana M.S., Almaraz M.C., Morcillo S., García Fuentes E., Rodríguez F., Rojo-Martinez G. (2012). Validación del FINDRISC (FINnish Diabetes Risk SCore) para la predicción del riesgo de diabetes tipo 2 en una población del sur de España. Estudio Pizarra. Med. Clínica.

[26] Federation International Diabetes (FID) (2024). Hiperglucemia Intermedia y la Diabetes de Tipo 2.

[27] Gomez-Arbelaez D., Alvarado-Jurado L., Ayala-Castillo M., Forero-Naranjo L., Camacho P.A., Lopez-Jaramillo P. (2015). Evaluation of the Finnish Diabetes Risk Score to Predict Type 2 Diabetes Mellitus in a Colombian Population: A Longitudinal Observational Study. World J. Diabetes.

[28] Carrillo-Larco R.M., Aparcana-Granda D.J., Mejia J.R., Bernabé-Ortiz A. (2020). FINDRISC in Latin America: A Systematic Review of Diagnosis and Prognosis Models. BMJ Open Diabetes Res. Care.

[29] Brito-Nuñez J., Cedeño-Rondón J., Pérez-Arciniega E., Brito-Núñez N. (2022). Riesgo de diabetes mellitus según el cuestionario Finnish Diabetes Risk Score (FINDRISC) en Indígenas Warao de Barrancas del Orinoco, Monagas, Venezuela. Rev. Salud Pública.

[30] Perez Bravo F.M. ¿Cómo Ayuda el Test de Ruffier-Dickson?. https://gaceta.cch.unam.mx/es/como-ayuda-el-test-de-ruffier-dickson.

[31] R: El Proyecto R Para Computación Estadística. https://www.r-project.org/.

[32] Kurani S.S., Lampman M.A., Funni S.A., Giblon R.E., Inselman J.W., Shah N.D., Allen S., Rushlow D., McCoy R.G. (2021). Association Between Area-Level Socioeconomic Deprivation and Diabetes Care Quality in US Primary Care Practices. JAMA Netw. Open.

[33] Khavjou O. (2025). Rural–Urban Disparities in State-Level Diabetes Prevalence Among US Adults, 2021. Prev. Chronic. Dis..

[34] MacDonald H., Papadopoulos A., Dewey C., Humphries S.A., Dodd W., Patel K., Little M. (2022). Sociodemographic Factors Associated with Knowledge of Type 2 Diabetes in Rural Tamil Nadu, India. Rural Remote Health.

[35] Viáfara-López C.A., Palacios-Quejada G., Banguera-Obregón A. (2021). Inequidad por la condición étnico-racial en el aseguramiento de salud en Colombia: Un estudio de corte transversal. Rev. Panam. Salud Pública.

[36] Puello E.C., Amador C.E., Luna J.M. (2016). Determinantes sociales de salud en los agricultores del resguardo indígena Zenú. Rev. Univ. Ind. Santander Salud.

[37] Nieto-Martinez R., Barengo N.C., Restrepo M., Grinspan A., Assefi A., Mechanick J.I. (2023). Large Scale Application of the Finnish Diabetes Risk Score in Latin American and Caribbean Populations: A Descriptive Study. Front. Endocrinol..

[38] Anaya González J.L., Perugachi Benalcazar I.A., Lechón Sandoval J.A., Velásquez Calderón C., Méndez Carvajal E.P., Silva Encalada C.M., Anaya González J.L., Perugachi Benalcazar I.A., Lechón Sandoval J.A., Velásquez Calderón C. (2025). Índice de masa corporal y probabilidad de diabetes tipo 2: Enfoque multiétnico en población Ecuatoriana. Más Vita Rev. Cienc. Salud.

[39] Hu S., Ji W., Zhang Y., Zhu W., Sun H., Sun Y. (2025). Risk Factors for Progression to Type 2 Diabetes in Prediabetes: A Systematic Review and Meta-Analysis. BMC Public Health.

[40] Cheran K., Murthy C., Bornemann E.A., Kamma H.K., Alabbas M., Elashahab M., Abid N., Manaye S., Venugopal S., Cheran K. (2023). The Growing Epidemic of Diabetes Among the Indigenous Population of Canada: A Systematic Review. Cureus.

[41] Mondal U.K., Ahmed K.Y., Thapa S., Kalinna B., Pak S.C., Anyasodor A.E., Mahmood S., Shiddiky M.J.A., Ross A.G. (2024). A Systematic Review of the Major Risk Factors for Type Two Diabetes among Aboriginal Australians. BMC Public Health.

[42] Ndetei D.M., Mutiso V., Musyimi C., Nyamai P., Lloyd C., Sartorius N. (2024). Association of Type 2 Diabetes with Family History of Diabetes, Diabetes Biomarkers, Mental and Physical Disorders in a Kenyan Setting. Sci. Rep..

[43] Zaki S., Alam M.F., Sharma S., El-Ashker S., Ahsan M., Nuhmani S. (2024). Impact of Concurrent Exercise Training on Cardiac Autonomic Modulation, Metabolic Profile, Body Composition, Cardiorespiratory Fitness, and Quality of Life in Type 2 Diabetes with Cardiac Autonomic Neuropathy: A Randomized Controlled Trial. J. Clin. Med..

[44] Wang X., Wu Y., Wang Y., Zhou J., Liu T. (2024). Relationship between Metabolically Healthy Overweight/Obesity and Risk of Type 2 Diabetes in Different Ethnicity: A Prospective Cohort Study in Southwest China. BMC Public Health.

[45] Singh S., Kriti M., K.S. A., Sarma D.K., Verma V., Nagpal R., Mohania D., Tiwari R., Kumar M. (2024). Deciphering the Complex Interplay of Risk Factors in Type 2 Diabetes Mellitus: A Comprehensive Review. Metab. Open.

[46] Nieto-Martínez R., González-Rivas J.P., Ugel E., Marulanda M.I., Durán M., Mechanick J.I., Aschner P. (2019). External Validation of the Finnish Diabetes Risk Score in Venezuela Using a National Sample: The EVESCAM. Prim. Care Diabetes.

[47] Yovera-Aldana M., Mezones-Holguín E., Agüero-Zamora R., Damas-Casani L., Uriol-Llanos B., Espinoza-Morales F., Soto-Becerra P., Ticse-Aguirre R. (2024). External Validation of Finnish Diabetes Risk Score (FINDRISC) and Latin American FINDRISC for Screening of Undiagnosed Dysglycemia: Analysis in a Peruvian Hospital Health Care Workers Sample. PLoS ONE.

